# Circulating miRNA-23b and miRNA-143 Are Potential Biomarkers for In-Stent Restenosis

**DOI:** 10.1155/2020/2509039

**Published:** 2020-09-16

**Authors:** Nicolás Saavedra, Gabriel Rojas, Jesús Herrera, Camilo Rebolledo, Jenny Ruedlinger, Luis Bustos, Braulio Bobadilla, Luis Pérez, Kathleen Saavedra, Tomás Zambrano, Fernando Lanas, Luis A. Salazar

**Affiliations:** ^1^Center of Molecular Biology & Pharmacogenetics, Department of Basic Sciences, Scientific and Technological Bioresource Nucleus, Universidad de La Frontera, Temuco, Chile; ^2^Department of Public Health, Faculty of Medicine, Universidad de La Frontera, Temuco, Chile; ^3^Department of Internal Medicine, Faculty of Medicine, Universidad de La Frontera, Temuco, Chile; ^4^Department of Internal Medicine, Faculty of Medicine, Universidad de Concepción, Concepción, Chile; ^5^Department of Medical Technology, Faculty of Medicine, Universidad de Chile, Santiago, Chile

## Abstract

In-stent restenosis (ISR) is one of the main complications in patients undergoing percutaneous coronary angioplasty, and microRNAs participate in the contractile-to-synthetic phenotypic switch of vascular smooth muscle cells, a hallmark of restenosis development. MicroRNAs (miRNAs) can be released into circulation from injured tissues, enticing a potential role as noninvasive biomarkers. We aimed to evaluate circulating levels of miRNA-23b, miRNA-143, and miRNA-145 as diagnostic markers of ISR. 142 patients with coronary artery disease undergoing successful angioplasty and a follow-up angiography were included. Subjects were classified according to the degree of obstruction at the angioplasty site into cases (≥50%) or controls (<50%). Total RNA was isolated from plasma to quantify circulating miRNAs levels, and the ROC curves were constructed. Among circulating miRNAs assessed, miRNA-23b and miRNA-143 were significantly lower in cases (miRNA-23b: 18.4x10^−5^ and miRNA-143: 13.7x10^−5^) than controls (miRNA-23b: 5.2x10^−5^, *p* < 0.0001; miRNA-143: 4.0x10^−5^, *p* < 0.0001). Plasma levels of miRNA-145 showed no significant differences. The analysis of the ROC curves showed an area under the curve for miRNA-23b of 0.71 (95% CI: 0.62-0.80, *p* < 0.0001) and 0.69 for miRNA-143 (95% CI: 0.60-0.78; *p* < 0.0001). Our data suggest that plasma levels of miRNA-23b and miRNA-143 could be useful as noninvasive biomarkers of ISR.

## 1. Introduction

Restenosis results from a reduced diameter of the lumen of a blood vessel following percutaneous coronary angioplasty (PCA). Angiographically, restenosis is defined dichotomously as a luminal narrowing of more than 50% in the follow-up angiography, which occurs as a consequence of arterial damage with subsequent neointimal tissue proliferation [1]. Although a reduction in the percentage of subjects developing restenosis has been observed at the same time PCA has evolved, in-stent restenosis (ISR) continues to be one of the main complications in patients undergoing this procedure [2]. Moreover, the uptake of percutaneous intervention in larger numbers of patients with increasingly complex lesion characteristics and disease comorbidities means that the number of patients presenting with restenosis remains considerable in absolute terms [3]. Several pathogenic mechanisms have been associated with restenosis development, among which we can find elastic recoil of the vessels, arterial remodeling, and neointimal hyperplasia. The two latter particularly related to ISR though a phenotypic change of the vascular smooth muscle cells (VSMCs) from a contractile to a proliferative state under the induction of platelet-derived growth factor (PDGF), resulting in accumulation of VSMC in the intimal arterial layer [4–6].

MicroRNAs (miRNAs) are short noncoding RNA molecules that negatively affect gene expression through mRNA cleavage or inhibition of protein translation, resulting in profound and complex regulatory networks [7]. In the cardiovascular system, miRNAs regulate basic functions in almost all cell types including cardiac muscle, endothelial cells, smooth muscle, inflammatory cells, and fibroblasts and, therefore, play a pivotal role in the pathogenesis of several cardiovascular diseases (CVDs). Numerous miRNAs have been implicated in the contractile-to-synthetic phenotypic switch of VSMC, including miRNA-143/145, miRNA-23b, miRNA-22, miRNA-133, miRNA-125a, miRNA-195, miRNA-663, miRNA-21, miRNA-221/222, miRNA-146a, miRNA-206, miRNA-181b, and miRNA-31, among others [8]. For instance, the miRNA-143/145 cluster coordinates the phenotypic modulation of VSMC through a cooperative network of transcription factors, such as Kruppel-Like Factor 5 (KLF5), Kruppel-Like Factor 4 (KLF4), and the potent transcriptional coactivator myocardin (MYOCD), to promote differentiation and suppress VSMCs proliferation [9, 10]. Thereby, deficiency of miRNA-145/143 promotes the synthetic phenotype of VSMCs. Furthermore, adenoviral-mediated gene transfer of miRNA-145/143, which downregulate the expression of this miRNAs after injury, inhibits neointimal lesion formation in injured rat carotid arteries [11]. On the other hand, miRNA-23b plays a critical role in angiogenesis, cardiac ischemia, and homeostasis of endothelial cells [12]. Moreover, the overexpression of miRNA-23b in balloon-injured arteries by Ad-miRNA-23b markedly decreased neointimal hyperplasia in VSMC, *in vitro* [13]. Yet, another relevant and highly attractive feature of miRNAs corresponds to their remarkable extracellular stability, an outcome that has received especial attention due to a promising role as blood-based biomarkers for diagnosis and prognosis of several illnesses [14]. Considering this background, we evaluated levels of circulating miRNA-23b, miRNA-143, and miRNA-145 as potential diagnostic markers of ISR in Chilean subjects with coronary artery disease (CAD).

## 2. Methods

### 2.1. Subjects

A diagnostic test study was designed using unpaired incident cases and controls, including 142 patients with CAD who underwent successful coronary artery angioplasty and follow-up coronary angiography after at least 6 months of stent implantation. Participants were classified in two groups: (1) case group, with stenosis ≥ 50% and (2) control group, with stenosis < 50%, a criterion concordant with the degree of obstruction at the angioplasty site. The obstruction was measured in the follow-up and was regarded as the gold standard. Demographic, anthropometric, and cardiovascular risk factors such as diabetes, hypertension, and dyslipidemia were recorded. Patients were invited to participate in the cardiology services of public Chilean hospitals, Dr. Hernán Henríquez Aravena Hospital (Temuco, Chile) and Dr. Guillermo Grant Benavente (Concepción, Chile). All subjects enrolled were included in this study after accepting their participation by signing a written informed consent approved by both, the Ethics Committees of University of La Frontera and Health Service of Concepción.

### 2.2. Sampling and RNA Extraction

For detection of circulating miRNAs, we obtained a sample of whole blood anticoagulated with EDTA at the time of the angiographic control using standard venipuncture blood extraction. Then, plasma was separated by centrifugation (15 minutes at 3.000 rpm) and transferred into nuclease-free tubes for subsequent storage at -80°C until RNA extraction procedures. Plasma RNA was isolated using the miRNeasy Mini Kit (Qiagen) following the supplemental protocol for RNA purification, including miRNAs. Prior to extraction, 5 *μ*L of the exogenous miRNA-39 (5 nM) of *Caenorhabditis elegans* (cel-miRNA-39) was added to 0.2 mL of plasma, for the normalization of technical variations (sample quality and extraction efficiency) between samples.

### 2.3. Circulating miRNAs

To quantify circulating miRNA-143, miRNA-145, and miRNA-23b levels from total RNA, a first strand of complementary DNA (cDNA) was synthesized using the TaqMan® Advanced miRNA (Applied Biosystems) synthesis kit, which polyadenylated the 3′ end and added an adaptor to the 5′ end of all miRNA sequences contained in the sample. Then, miRNA detection was completed by quantitative polymerase chain reaction (qPCR), using the TaqMan® Advanced miRNA Assays for each selected miRNA (Thermo Fisher Scientific assay IDs 478713_mir, 477915_mir and 477991_mir). These assays were performed in a StepOne™ Real-Time PCR System (Applied Biosystems) using the following thermocycling protocol: 20 seconds at 95°C and 40 cycles of 95°C for 1 second and 60°C for 20 seconds. All qPCR assays were performed in duplicate, and miRNAs quantification was obtained by the 2^-*Δ*Ct^ method, using cel-miRNA-39 to normalize miRNA expression.

### 2.4. Statistical Analysis

The statistical analysis was carried out with the software SPSS 25.0 (SPSS, Inc., Chicago, USA). The unpaired two-tailed *t*-test or its nonparametric equivalent (Mann–Whitney *U* test) was used to assess differences between demographic and biometric characteristics and circulating miRNA quantification between cases and controls. The comparisons between dichotomous variables were analyzed using the chi-squared test. The receiver operating characteristic (ROC) curves were generated to evaluate the diagnostic capacity of each circulating miRNA, using the area under the curve (AUC) to determine cutoff values that maximizes sensitivity and specificity. A value of *p* < 0.05 was defined as statistically significant.

## 3. Results

### 3.1. Clinical Characteristics of Subjects

Clinical and anthropometric characteristics of the participants are shown in Table 1. No significant differences were observed between age (*p* = 0.640), body mass index (BMI), systolic blood pressure (SBP), diastolic blood pressure (DBP), heart rate, diabetes, dyslipidemia, hypertension, and obesity. However, a higher percentage of men was observed in the case group (*p* = 0.042), as well as a higher percentage of subjects treated with bare-metal stent (BMS) in the same group (*p* = 0.002).

### 3.2. Circulating miRNAs and ROC Curves

**Figure 1:**
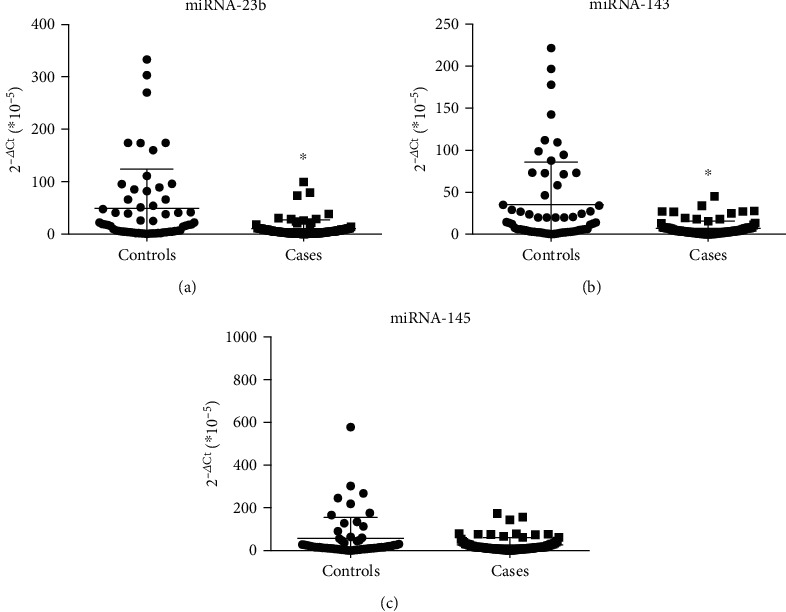
Quantification of miRNAs: (a) miRNA-23b, **(**b) miRNA-143, and (c) miRNA-145 in plasma samples of cases and controls.

**Figure 2:**
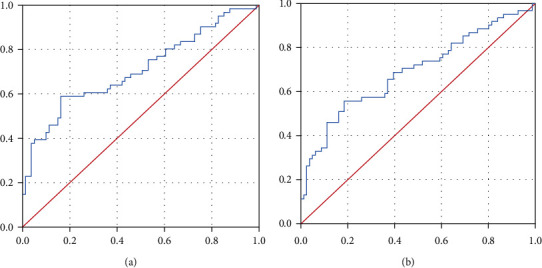
Analysis of the ROC curves showing the sensitivity (*Y* axis) and 1-specificity (*X* axis) for (a) miRNA-23b and (b) miRNA-143 as potential diagnostic markers for ISR.

Plasma levels of circulating miRNA-143 and miRNA-23b were significantly lower in the case group compared to controls (*p* < 0.001). Circulating miRNA-145 levels did not show differences between both groups (*p* = 0.099) (Figure 1). Based on these results, we therefore selected miRNA-23b and miRNA-143 to evaluate their diagnostic potential through the ROC curve analysis. As shown in Figure 2, the ROC curve for miRNA-23b showed an AUC of 0.71 (95% CI, 0.620-0.798, p <0.001), while the ROC curve for miRNA-143 displayed an AUC of 0.692 (95% CI, 0.601-0.782, *p* < 0.001). We then established the most appropriate cutoff point for both tests, considering the best performance for specificity and sensitivity, resulting in 14.3 x 10^−5^ (miRNA-23b) and 11.4 x 10^−5^ (miRNA-143). Sensitivity, specificity, positive predictive value (PPV), and negative predictive value (NPV) are shown in Table 2. In addition, by combining measures from both miRNAs, the ROC analysis showed superior sensitivity (0.82), whereas specificity reached 0.68 (Table 2).

## 4. Discussion

In the present study, we evaluated circulating levels of miRNA-23b, miRNA-143, and miRNA-145 in patients undergoing successful coronary arterial angioplasty, with a follow-up coronary angiography after at least 6 months since stent implantation, in order to identify differences in miRNA levels between patients who developed ISR versus controls.

ISR is one of the most relevant complications after stent placement and is characterized by the phenotypic switch of VSMC. Different reports indicate that miRNAs play a significant role in this process, for instance, miRNA-143, miRNA-145, and miRNA-23b maintain a contractile phenotype in VSMCs of healthy blood vessels [9, 10, 15–19]; therefore, circulating miRNAs could reflect cellular processes occurring in the blood vessels after stent implantation [16, 20]. Moreover, due to miRNAs can be found in several body fluids, their potentials to identify different pathologies have been widely explored in CVD such as acute myocardial infarction [21], CAD [22], and stroke [23]. In the case of ISR, we here show that miRNA-23b could be used as a noninvasive biomarker to identify patients with ISR. This miRNA is highly expressed in cell cultures of VSMC, decreasing their proliferation, differentiation, and migration [13]. In addition, *in vitro* studies show that miRNA-23b regulates the expression of the Forkhead Box protein (Fox04) [24], which represses differentiation of smooth muscle cells and activates the migration of VSMC in response to TNF-*α* and urokinase-type plasminogen activator (uPA) [15, 19] through the MEK/ERK signaling pathway [25] and SMAD3 [19], a protein important for the proliferation of VSMC [26]. These data could explain the decreased levels of miRNA-23b detected in the plasma of patients with ISR.

Similarly, studies have reported AUC values of 0.839 for miRNA-143 and 0.871 for miRNA-145, in addition to high specificity and sensitivity (0.9 and 0.7), respectively [27]. We also observed a repression of miRNA-143 in subjects who develop ISR. Apparently, the most important trigger of miRNA-143 repression is PDGF, a key element in the mediation of vascular response to injury acting through the protooncogene tyrosine-protein kinase SRC and phosphoprotein p53, with the net result of promoting both proliferation and migration of VSMC [16]. A significant difference in plasma levels of miRNA-143 in restenosis of vascular occlusive disease of the lower extremities has also been reported, with significantly decreased expression [28].

The availability of noninvasive biomarkers with high resolution for specific biological processes could add significant incremental value to the clinical arsenal currently available for short-term follow-up of patients treated with PCA, which could reduce the number of candidates for invasive angiographic control [29]. Identification of differential miRNAs and their potential use as biomarkers in patients with ISR still requires greater consistency. Moreover, miRNAs are extremely stable in plasma/serum and resistant to some hard conditions including low and high pH, boiling, and long storage and can withstand repetitive freezing and thawing cycles [30]. We also highlight the need for longitudinal studies depicting changes in miRNA levels from the first stages of the intervention. Currently, incipient efforts have been reported to a promissory use of miRNAs for stent intervention in animal models, which holds potential for personalized therapy [31, 32].

In conclusion, our data show decreased levels of miRNA-23b and miRNA-143 in the plasma of subjects with ISR versus controls, a differential result that could confer these miRNAs a potential use as noninvasive biomarkers for ISR.

## Figures and Tables

**Table 1:** Clinical and anthropometric characteristics of cases and controls.

Parameter	Controls (*n* = 61)	Cases (*n* = 81)	*p* value
Age (years)	67.0 ± 9.3	64.1 ± 10.3	0.640
BMI (kg/m^2^)	27.6 ± 4.2	28.2 ± 3.9	0.326
SBP (mmHg)	130.7 ± 25.9	130.4 ± 23.6	0.942
DBO (mmHg)	69.5 ± 12.2	73.1 ± 12.1	0.081
HR (bpm)	68.6 ± 11.0	71.0 ± 12.2	0.301
Male (%)	66.7	80.5	0.042
BMS (%)	65.8	86.4	0.002
Diabetes mellitus (%)	28.0	41.0	0.091
Dyslipidemia (%)	69.3	74.4	0.489
Hypertension (%)	84.2	85.9	0.769
Obesity (%)	26.3	27.4	0.870
Lesion vessel (%)			
LMCA	1.1	1.1	—
LAD	47.5	44.8	—
CX	18.3	31.7	—
RCA	33.1	22.4	—
Diameter (mm)	3.1 ± 0.3	2.9 ± 0.1	0.673
Length (mm)	22.3 ± 4.1	22.6 ± 3.4	0.710

Values expressed as mean ± standard deviation. BMI: body mass index; SBP: systolic blood pressure; DBP: diastolic blood pressure; HR: heart rate; ISR: in-stent restenosis; BMS bare-metal stent; LMCA: left main coronary artery; LAD: left anterior descending artery; CX: circumflex artery; RCA: right coronary artery.

**Table 2:** Diagnostic characteristics for plasma miRNA-23b and miRNA-143.

miRNAs	Sensitivity	Specificity	PPV	NVP
miRNA-23b	0.59	0.84	0.83	0.61
miRNA-143	0.56	0.82	0.80	0.58
miRNA-23b/miRNA-143	0.82	0.68	0.77	0.73

PPV: positive predictive value; NPV: negative predictive value.

## Data Availability

The data and materials in the current study are available from the corresponding author on reasonable request.
